# M-wave changes caused by brief voluntary and stimulated isometric contractions

**DOI:** 10.1007/s00421-023-05228-x

**Published:** 2023-05-19

**Authors:** Javier Rodriguez-Falces, Armando Malanda, Javier Navallas, Nicolas Place

**Affiliations:** 1 Department of Electrical and Electronical Engineering, Public University of Navarra, Campus de Arrosadía s/n. 31006, Pamplona, Spain; 2 Institute of Sport Sciences, University of Lausanne, Lausanne, Switzerland

**Keywords:** Muscle shortening, Fascicle length, Fiber diameter, Isometric contraction, Tetanic stimulation, Muscle bulging

## Abstract

**Introduction:**

Under isometric conditions, the increase in muscle force is accompanied by a reduction in the fibers’ length. The effects of muscle shortening on the compound muscle action potential (M wave) have so far been investigated only by computer simulation. This study was undertaken to assess experimentally the M-wave changes caused by brief voluntary and stimulated isometric contractions.

**Methods:**

Two different methods of inducing muscle shortening under isometric condition were adopted: (1) applying a brief (1 s) tetanic contraction and (2) performing brief voluntary contractions of different intensities. In both methods, supramaximal stimulation was applied to the brachial plexus and femoral nerves to evoke M waves. In the first method, electrical stimulation (20 Hz) was delivered with the muscle at rest, whereas in the second, stimulation was applied while participants performed 5-s stepwise isometric contractions at 10, 20, 30, 40, 50, 60, 70, and 100% MVC. The amplitude and duration of the first and second M-wave phases were computed.

**Results:**

The main findings were: (1) on application of tetanic stimulation, the amplitude of the M-wave first phase decreased (~ 10%, *P* < 0.05), that of the second phase increased (~ 50%, *P* < 0.05), and the M-wave duration decreased (~ 20%, *P* < 0.05) across the first five M waves of the tetanic train and then plateaued for the subsequent responses; (2) when superimposing a single electrical stimulus on muscle contractions of increasing forces, the amplitude of the M-wave first phase decreased (~ 20%, *P* < 0.05), that of the second phase increased (~ 30%, *P* < 0.05), and M-wave duration decreased (~ 30%, *P* < 0.05) as force was raised from 0 to 60–70% MVC force.

**Conclusions:**

The present results will help to identify the adjustments in the M-wave profile caused by muscle shortening and also contribute to differentiate these adjustments from those caused by muscle fatigue and/or changes in Na^+^–K^+^ pump activity.

## Introduction

It is known that, under isometric conditions, muscle fascicle length shortens when muscle force is increased (Narici et al. 1996; Hodges et al. 2003). Muscle fascicle shortening occurs more markedly at low forces (Hodges et al. 2003) when tendon stiffness is low, which implies that small increases in force result in large reductions in fascicle length (Bennett et al. 1986; Shadwick 1990). The reduction in fiber length gives rise to a cascade of events in the muscle. First, it produces an increase in the diameter of individual muscle fibers (Aidley 1998). Moreover, to accommodate the decrease in fiber length and the augmentation of fiber diameter, the muscle increases in thickness, the so-called muscle bulging (Azizi et al. 2008). All the above structural events have profound implications in the electromyographic (EMG) potentials recorded by the electrode. At the level of individual fibers, an increase in fiber diameter causes a faster conduction of action potentials (Håkansson 1956; Morimoto 1986). The combination of an increased conduction velocity and a decreased fiber length leads to single fiber action potentials with shorter duration (Rodriguez-Falces and Place 2014). Muscle bulging modifies not only the characteristics of the volume conductor (geometry and conductivity), but also the EMG recording conditions, such as the distance from the electrode to the muscle fibers (Mesin et al. 2006). Therefore, to investigate the effects of muscle shortening on EMG potentials, it is important to consider not only the reduction in fascicle length, but also the changes in the geometrical and physiological characteristics of the muscle caused by this shortening.

The compound muscle action potential (M wave) is one of the most utilized tools to study the changes in sarcolemmal membrane excitability. The M wave comprises two phases with a different electrical origin: the first “propagating” phase is generated by the propagation of the intracellular action potentials along the fibers, the second “non-propagating” phase reflects the extinction of these action potentials at the tendon (Rodriguez-Falces et al. 2022). Despite the importance of the M wave, no experimental study has been conducted to characterize the effects of muscle shortening on the M-wave profile. These shortening effects have, so far, only been investigated by computer simulation studies. Specifically, two EMG models have been proposed. The first one is a complex finite-element model, which included changes in the shape and conductivity of the volume conductor with shortening (Mesin et al. 2006). This model revealed two main effects: (1) as the muscle shortens and bulges, the fibers increase their distance from the detection electrode, which results in a decrease of the amplitude of the propagating component; (2) as the muscle shortens, the tendon endings approach the electrodes, thus causing an enlargement of the non-propagating component. Our group proposes a second analytical model, which confirmed the findings of Mesin and colleagues (Rodriguez-Falces and Place 2014; Rodriguez-Falces et al. 2022). In addition to the adjustments in amplitude, our model showed that muscle shortening also provokes a decrease in the duration of the M wave (Rodriguez-Falces et al. 2022). In the present study, the above simulation results were verified using real M waves evoked at different muscle lengths.

Knowledge on how the M-wave properties are influenced by muscle length is important to correctly interpret changes in the M wave during and after muscle contractions. Indeed, alterations in M-wave properties are generally solely attributed to changes in ionic concentrations across the membrane due to muscle fatigue and/or Na^+^–K^+^ pump activity, while part of these M-wave adjustments may be due to changes in muscle fiber length. For example, it has been shown that, after a brief voluntary contraction, the amplitude of the M-wave second phase increases and M-wave duration decreases for a brief period (~ 15 s) (Hicks et al. 1989; Rodriguez-Falces et al. 2015). These short-term changes in the M wave were interpreted by McComas et al (1994) as being caused by the enhancement of the electrogenic Na^+^–K^+^ pump: however, our previous research indicates that these M-wave changes could be due to the fact that muscle fiber length remains shortened for a few seconds after a contraction (Rodriguez-Falces and Place 2017a). Another example is that, during a sustained maximal voluntary contraction, the amplitude of the M-wave second phase increased initially for the first ~ 30 s and then decreased for the following ~ 60 s (Rodriguez-Falces and Place 2017b). One possible interpretation for the later depression of the second phase is that, as the maximal contraction is sustained beyond 30 s, muscle force declines steeply, which allows muscle fascicle length to increase. Therefore, an experimental characterization of the muscle shortening effects on the M-wave profile will help to differentiate the adjustments in the M wave caused by changes in muscle length from those caused by muscle fatigue and/or changes in Na^+^–K^+^ pump activity.

The objective of the present study was to assess experimentally the effects of brief isometric muscle contractions (both stimulated and voluntary) on the M-wave characteristics. Based on previous simulation studies (Mesin et al. 2006; Rodriguez-Falces et al. 2022), we hypothesized that, as the muscle shortens, the amplitude of the M-wave second phase increases markedly, the first phase diminishes moderately, and the M-wave duration decreases. To test the muscle at different lengths under isometric conditions, we adopted two different approaches. The first approach was implemented using repetitive stimulation and is based on the idea that, if the frequency of stimulation is sufficiently high (> 10 Hz), the muscle undergoes progressive shortening at the beginning of the stimulation (Chang and Shields 2002). The second strategy to test the muscle at varying lengths was implemented by performing brief voluntary muscle contractions at different intensities and delivering an electrical stimulus on the ongoing contraction. Because the first method of inducing muscle shortening is based on electrically elicited contractions, one may argue that this is an “artificial” method; for this reason, a second alternative method where muscle shortening is achieved using voluntary contractions is adopted. Finally, it is possible that some sliding between the recording electrodes and the muscle underneath occurs during isometric contractions. Because the degree of sliding is supposed to be lower in pennate than in fusiform muscles, a representative of each muscle architecture, vastus lateralis and biceps brachii, was examined.

## Materials and methods

### Participants

Fifteen participants (6 women) aged between 21 and 28 years (mean ± SD: 23.3 ± 2.1 years) with no previous neuromuscular or musculoskeletal disorders participated in the study. Their average height and body mass were 176 ± 5 cm and 68.2 ± 5.3 kg, respectively. The experiments were conducted in accordance with the Declaration of Helsinki and were approved by the research ethics board of the Public University of Navarra, Spain (PI-010/21). Written informed consent was obtained from all participants prior to the study initiation.

### Experimental setup

The muscle shortening effects were investigated in M waves recorded in the *vastus lateralis* and *biceps brachii*. During the recordings on the *vastus lateralis*, participants were seated comfortably on a custom-built chair with the trunk–thigh angle at 100°. The knee angle was 90°. Possible movements of the upper body were minimized by two crossover shoulder harnesses and a belt across the lower abdomen. Quadriceps force was recorded using a strain gauge (STS, SWJ, China, linear range: 0–2452 N, sensitivity 2 mV/V and 0.0017 V/N) that was attached to the chair and securely strapped to the ankle with a custom-made mold. During the recordings on the *biceps brachii*, participants were seated with the elbow flexed at 120°, while the shoulder was 90° abducted. The forearm was vertical and supinated, while the hand was holding an adjustable handle connected to a strain gauge with the same characteristics as that used for the quadriceps. The force signal (for both knee extension and elbow flexion) was sampled at 1000 Hz using an analog-to-digital conversion system (MP150; BIOPAC, Goleta, CA, USA).

### Localization of the innervation zone

The location of the innervation zone was determined in each muscle using a dry linear array of 16 electrodes (inter-electrode distance, 5 mm) during gentle isometric contractions. The array was connected to a multichannel amplifier (OT Bioelettronica, Torino; bandwidth 10–500 Hz). Surface EMG signals were monitored during low-intensity voluntary contractions (15–30% MVC) using a single-differential (bipolar) configuration. The position of the innervation zone was that corresponding to the channel of the array showing phase reversal or minimum amplitude (Masuda et al. 1985).

### Electromyographic recordings

Surface EMG potentials were recorded using self-adhesive Ag/AgCl surface electrodes (Kendall Meditrace 100), with circular shape (recording diameter 10 mm). Before the electrodes were placed, the skin was shaved, abraded, and cleaned with rubbing alcohol to minimize the impedance at the skin–electrode interface. Surface EMG signals were amplified (gain: 500 V/V, bandwidth: 10–5000 Hz) and digitized (sampling frequency of 5000 Hz) using an analog-to-digital conversion system (MP150; BIOPAC, Goleta, CA). Subsequently, a second-order Butterworth 10–1000 Hz was applied to the signal.

In both the *vastus lateralis* and *biceps brachii*, the recording electrodes were arranged in a “belly–tendon” configuration. Specifically, the “belly” electrode was located over the innervation zone of each muscle (see above). The “tendon” electrode corresponding to the *vastus lateralis* was placed over the patellar tendon, whereas the “tendon” electrode associated to the *biceps brachii* was placed over the flexor retinaculum of the wrist. The ground electrode was placed adjacent to the tendon electrode in both muscles. The reason why the belly–tendon configuration was adopted was because this montage allows to record the entire content of the electrical potential generated by the muscle (including the generation, propagation, and extinction events), and hence, the muscle shortening effects would be manifested to their full extent and authenticity with this electrode configuration (Rodriguez-Falces and Place 2016).

### Stimulation procedure

The femoral nerve was stimulated using a self-adhesive cathode (5 cm diameter; Dermatrode, American Imex, Irvine, CA, USA) placed in the femoral triangle, 3–5 cm below the inguinal ligament. The anode was a large (5 × 10 cm) rectangular self-adhesive electrode (Compex, Ecublens, Switzerland) located over the gluteal fold. The brachial plexus was stimulated using a self-adhesive cathode (1 cm diameter, Kendall Meditrace 100) placed in the supraclavicular fossa, whereas the anode (5 × 10 cm, Compex) was positioned on the acromion (Smith et al. 2007). Single rectangular pulses were delivered by a high-voltage constant current stimulator (DS7AH; Digitimer, Hertfordshire, UK): pulse width was set at 1 ms for femoral nerve stimulation (Neyroud et al. 2013), and at 0.1 ms for brachial plexus stimulation (Smith et al. 2007). In each muscle, the stimulus intensity corresponding to full motor unit recruitment was determined by gradually increasing the stimulus intensity until a plateau in the M-wave amplitude was observed. This level of intensity was then further increased by 20% to ensure that the stimulation remained supramaximal throughout the experimental session (Rodriguez-Falces and Place 2016).

### Experiment 1: M waves at the beginning of a tetanic stimulation

The first approach to test the muscle at different lengths was to apply repetitive stimulation at a frequency sufficiently high to produce a tetanic contraction. If this condition is satisfied, the muscle will undergo progressive shortening at the beginning of the stimulation. The reason for this shortening is that the first stimuli of a tetanic train occur before maximal tetanic force has been generated, when the tendon’s stiffness is low, and thus small increases in force results in large decreases in muscle fascicle length (Hodges et al. 2003). As a result, the successive stimuli at the beginning of a tetanic train would “test/catch” the muscle at progressively shorter lengths.

**Fig. 1:**
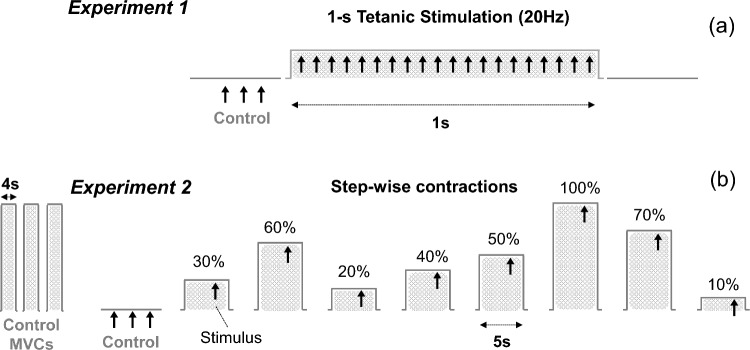
Two experimental protocols utilized to test the muscle at different lengths. In the first protocol (**a**), a train of tetanic stimulation at 20 Hz was delivered to the femoral nerve and brachial plexus. In the second protocol (**b**), participants were required to perform stepwise contractions at different percentages of the maximal voluntary contraction (MVC) in the vastus lateralis and biceps brachii and a supramaximal stimulus was applied toward the end of each contraction (vertical arrow)

The experiment consisted on delivering a burst of supramaximal electrical stimulation to the femoral nerve and brachial plexus at 20 Hz for 1 s (Fig. 1a). Three control supramaximal stimuli were delivered before the tetanic train. The choice of the 20 Hz stimulation frequency satisfied two requirements: first, it is high enough to produce a tetanic contraction (Cupido et al. 1996), and second, it is low enough to allow each M wave to develop fully between consecutive stimuli. The choice of such short duration of stimulation (1 s) was due to two reasons: first, to avoid action potential fatigue and membrane depolarization (Hanson 1974; Lannergren and Westerblad 1987), and also, to minimize pain in participants. Finally, the decision to stimulate the nerve trunk instead of stimulating the belly of the muscle (“over-the-muscle” stimulation) was to ensure complete recruitment of motor units at supramaximal current intensity (Gregory and Bickel 2005).

### Experiment 2: M waves superimposed on brief voluntary contractions of different intensities

The second approach to examine the muscle at varying lengths was to superimpose a single electrical stimulus on muscle contractions of different intensities (Fig. 1b). The rationale behind this strategy is that as voluntary force increases, the tendon elongates, thus allowing the muscle to operate at a shorter length (Hodges et al. 2003).

The experiment started with the assessment of participants’ maximal voluntary force. To do so, participants were asked to perform three brief (4 s) control MVCs (with 3 min of rest in between) and the average peak force from these three MVCs was calculated to yield an estimate of each individual’s maximal voluntary force. Subsequently, the participants were required to perform stepwise isometric contractions of 5 s at different contraction intensities: 10, 20, 30, 40, 50, 60, 70, and 100% of MVC force (Fig. 1b). To do this, the target force level for each contraction intensity was digitally displayed on a computer monitor in front of the participant. The order of contraction intensity was randomized and a resting period of 2 min was allowed between contractions. A single supramaximal stimulus was delivered to the femoral nerve and brachial plexus (superimposed on the ongoing contraction) at 3.5 s from the onset of each contraction. Three control supramaximal stimuli were delivered at rest before the contractions.

### Data analysis

For each M-wave potential, the amplitude and duration of the first (Ampli_FIRST_ and Dur_FIRST_) and second (Ampli_SECOND_ and Dur_SECOND_) phases were computed (for a visual definition of these parameters, see Rodriguez-Falces and Place 2017a). The onset for Dur_FIRST_ was determined by a deviation greater than 2 SDs of the baseline noise from the baseline, whereas the end point corresponded to the baseline-crossing point. This crossing point marked the onset of the second phase. The end-point for Dur_SECOND_ was determined by a deviation less than 2 SDs of the baseline noise from the baseline. The peak-to-peak amplitude (Ampli_PP_) was computed as the sum of Ampli_FIRST_ and Ampli_SECOND_. Peak-to-peak duration (Dur_PP_) was computed as the time interval between the first and second peaks of the M wave.

The above M-wave parameters were calculated using custom-written scripts implemented in Matlab (Mathworks, Natick MA). In the first experiment, the average of the control M waves (elicited at rest) was calculated, and all the M-wave parameters recorded during the tetanic train were expressed as percentage of the control responses. In the second experiment, all M-wave parameters were expressed relative to the control M waves elicited at rest.

### Statistics

Kolmogorov–Smirnov tests confirmed that each parameter analyzed in the current study was normally distributed. Sphericity of the data was verified before performing statistical analysis (Mauchly's test, *P* > 0.05). The changes in M-wave parameters during the 1-s tetanic train were investigated with a one-way repeated-measures ANOVA [Nº of response (1, 2, 3,…, 20)] for the *vastus lateralis* and *biceps brachii* separately. To examine the effects of contraction intensity on the superimposed M wave, a one-way repeated-measures ANOVA [contraction force (10, 20, 30, 40, 50, 60, 70, and 100% of MVC force)] was performed for the *vastus lateralis* and *biceps brachii* separately. When main effects were significant, Student–Newman–Keuls post hoc tests were conducted. Significance was set at *P* < 0.05. Data are presented as mean ± SD in the text and tables and as mean ± SE in the figures.

## Results

### Experiment 1: M waves at the beginning of a tetanic stimulation

**Fig. 2:**
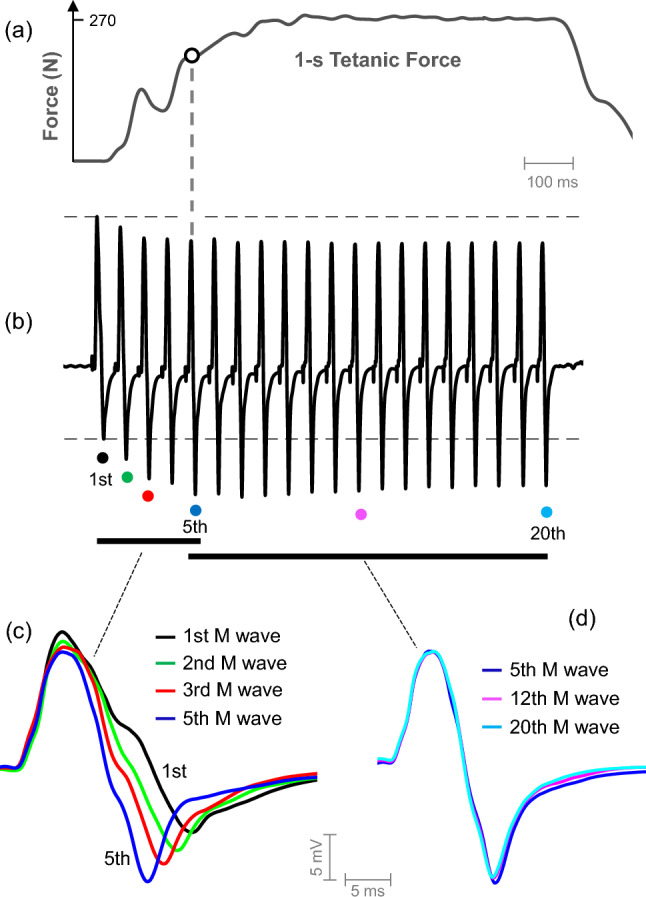
Representative example of a train of M waves obtained from the vastus lateralis during 1 s of tetanic stimulation at 20 Hz (**b**), together with the tetanic force produced (**a**). To better visualize the changes in the duration of the M wave, some selected responses are plotted, in a superimposed fashion, in (**c**) and (**d**). The stimulus artifact has been largely removed for demonstration purposes

Figure 2 shows a representative train of M waves recorded in one participant during 1 s of tetanic stimulation of the femoral nerve, together with the tetanic force produced. It can be seen that Ampli_FIRST_ decreased across the first five responses and then leveled off (plot b). Conversely, Ampli_SECOND_ increased markedly during the first five responses and then stabilized for the subsequent potentials (plot b). M-wave duration decreased pronouncedly during the first five M waves (plot c), but it remained stable for the subsequent potentials (plot d). Noteworthy, the M-wave parameters stopped changing at the fifth response, at a moment when the tetanic force was only ~ 70% of the maximal tetanic force (see vertical dashed line). It is also worth mentioning that the changes in Ampli_FIRST_, Ampli_SECOND_, and M-wave duration occurred in parallel (Fig. 2c).

**Fig. 3:**
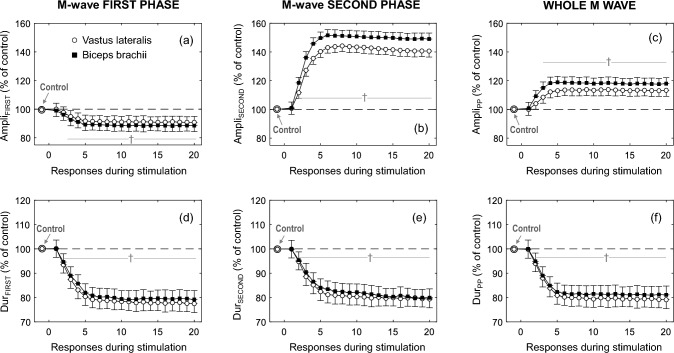
Time course of changes in amplitude and duration for the first phase (Ampli_FIRST_ and Dur_FIRST_, respectively) and the second phase (Ampli_SECOND_ and Dur_SECOND_, respectively) of the M wave, and also for the whole M wave (Ampli_PP_ and Dur_PP_, respectively), recorded in the vastus lateralis and biceps brachii during 1 s of tetanic stimulation of the femoral nerve and brachial plexus, respectively. All data are expressed as percentage of control values and reported as mean ± SE (*n* = 15). †Significant difference with control for all muscles (*p* < 0.05)

Figure 3 shows the average changes in the amplitude and duration parameters of the first phase (first column) and second phase (second column) of the M wave during the 1-s tetanic stimulation for the whole study group for the *vastus lateralis* and *biceps brachii*. It can be seen that, for both muscles, Ampli_FIRST_ decreased significantly across the first five responses of the train and then plateaued (Fig. 3a, *P* < 0.05, Table 1), whereas Ampli_SECOND_ increased during the first five M waves before stabilizing (Fig. 3b, *P* < 0.05, Table 1). The overall M-wave amplitude (Ampli_PP_) also increased across the first five M waves (Fig. 3c, *P* < 0.05). All duration parameters examined (Dur_FIRST_, Dur_SECOND_, and Dur_PP_) decreased significantly across the first five responses (*P* < 0.05, Table 1) and stabilized subsequently (Figs. 3d–f). The maximal force attained during the tetanic contraction in the *vastus lateralis* and *biceps brachii* was 45 ± 11% and 42 ± 10% of MVC force, respectively.

**Table 1:** Average values of amplitude and duration of the first and second phases of the M wave recorded in the vastus lateralis (VL) and biceps brachii (BB) during 1 s of tetanic stimulation of the femoral nerve and brachial plexus, respectively

		Response during the tetanic train							
Parameter	Muscle	Control values	1st	2nd	3rd	4th	5th	10th	20th
Ampli_FIRST_ (mV)	VL	12.9 ± 3.4	12.9 ± 3.3	12.7 ± 3.2	12.4 ± 3.4*	12.1 ± 3.3*	11.8 ± 3.1*	11.8 ± 3.2*	11.7 ± 3.1*
	BB	15.0 ± 3.6	15.0 ± 3.5*	14.6 ± 3.4*	14.1 ± 3.6*	13.7 ± 3.5*	13.4 ± 3.3*	13.4 ± 3.5*	13.4 ± 3.6*
Ampli_SECOND_ (mV)	VL	7.3 ± 2.2	7.3 ± 2.2	8.1 ± 2.7*	9.2 ± 2.8*	9.7 ± 2.7*	10.1 ± 2.9*	10.2 ± 2.6*	10.2 ± 3.0*
	BB	14.7 ± 3.8	14.7 ± 4.2	16.3 ± 4.0*	19.1 ± 4.1*	20.5 ± 4.4*	22.0 ± 4.3*	21.9 ± 4.0*	21.8 ± 4.2*
Dur_FIRST_ (ms)	VL	11.4 ± 2.8	11.5 ± 2.7	10.8 ± 2.9*	10.2 ± 2.8*	9.6 ± 2.7*	9.2 ± 2.5*	9.1 ± 2.6*	9.1 ± 2.5*
	BB	10.2 ± 2.1	10.2 ± 2.1	9.7 ± 1.4*	9.1 ± 1.3*	8.5 ± 1.7*	8.0 ± 1.7*	8.0 ± 1.7*	8.0 ± 1.7*
Dur_SECOND_ (ms)	VL	28.4 ± 9.7	28.3 ± 9.8	27.0 ± 9.6*	25.4 ± 9.4*	24.0 ± 9.7*	22.9 ± 9.1*	22.8 ± 9.3*	22.7 ± 9.0*
	BB	31.0 ± 10.2	31.0 ± 10.2	29.4 ± 9.9*	27.9 ± 9.3*	26.4 ± 9.0*	25.3 ± 9.6*	25.0 ± 9.5*	24.8 ± 9.3*

All values are expressed as mean ± SD

*Significantly different from control values (*P* < 0.05, *n* = 15)

### Experiment 2: M waves superimposed on voluntary contractions of different intensities

**Fig. 4:**
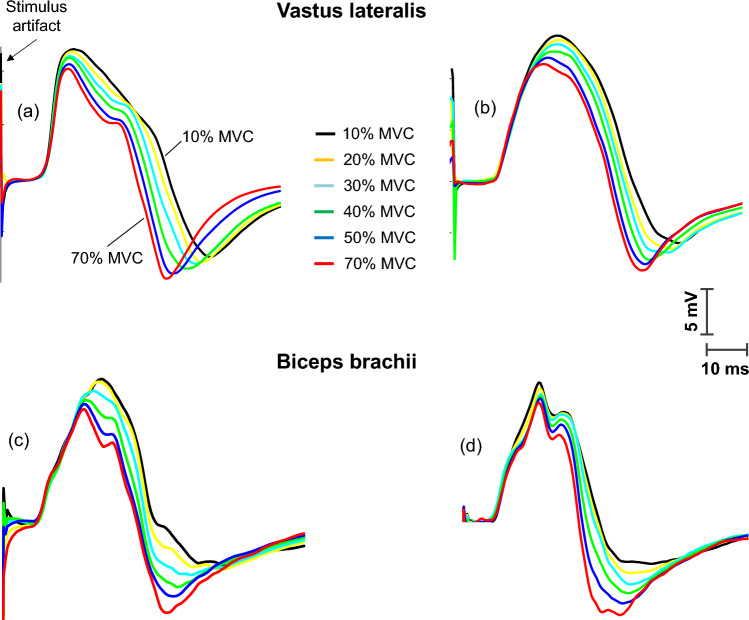
Representative examples of sets of M waves recorded in four participants, each set comprising M waves elicited during brief muscle contractions of different force levels [% of maximal voluntary contraction (MVC) force] in the vastus lateralis and biceps brachii

Figure 4 shows representative examples of sets of M waves recorded in four participants, each set containing M waves superimposed on muscle contractions of different intensities in the *vastus lateralis* (upper panel) and *biceps brachii* (bottom panel). It can be seen that, for both muscles, Ampli_FIRST_ decreased, Ampli_SECOND_ increased, and M-wave duration decreased as the contraction force increased. Also noteworthy is that the changes in Ampli_FIRST_, Ampli_SECOND_, and M-wave duration occurred concurrently.

**Fig. 5:**
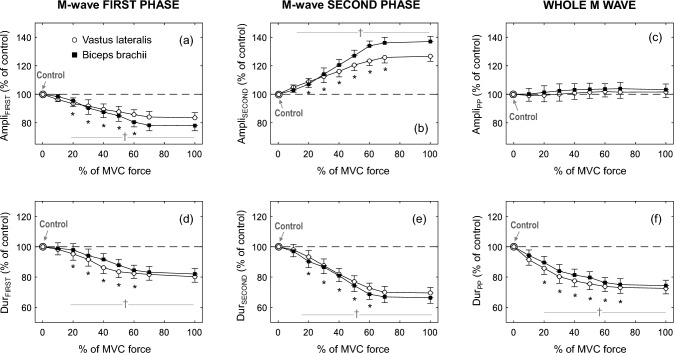
Group data of amplitude and duration for the first phase (Ampli_FIRST_ and Dur_FIRST_, respectively) and the second phase (Ampli_SECOND_ and Dur_SECOND_, respectively) of the M wave, and also for the whole M wave (Ampli_PP_ and Dur_PP_, respectively), elicited during brief muscle contractions of different force levels [% of maximal voluntary contraction (MVC) force] in the vastus lateralis and biceps brachii. All data are expressed as percentage of control values and reported as mean ± SE (*n* = 15). †Significant difference with control for all muscles (*P* < 0.05). *Significantly different from the preceding contraction intensity

Figure 5 shows the average values of amplitude and duration of the first phase (first column) and second phase (second column) of the M waves superimposed on muscle contractions of different intensities. It can be seen that, for both muscles, Ampli_FIRST_ decreased significantly as contraction force increased from 0 to 60% MVC, and then leveled off at higher force levels (Fig. 5a, *P* < 0.05, Table 2). In contrast, Ampli_SECOND_ increased with force level up to 70% MVC and then remained stable at higher force levels (Fig. 5b, *P* < 0.05, Table 2). The overall M-wave amplitude (Ampli_PP_) remained unchanged with increasing force level (Fig. 5c, *P* < 0.05). All duration parameters examined (Dur_FIRST_, Dur_SECOND_, and Dur_PP_) decreased with contraction intensity up to 60–70% MVC and then plateaued for higher intensities (Fig. 5d–f, *P* < 0.05).

**Table 2:** Average values of amplitude and duration of the first and second phases of the M wave elicited during brief muscle contractions of different force levels in the vastus lateralis (VL) and biceps brachii (BB)

Parameter	Muscle	Force level (% maximal voluntary contraction force)							
		Control values	10%	20%	30%	40%	50%	60%	100%
Ampli_FIRST_	*VL*	12.9 ± 3.2	12.6 ± 3.1	12.3 ± 3.4*	12.0 ± 3.4*	11.6 ± 3.1*	11.3 ± 3.2*	11.1 ± 3.2*	10.9 ± 3.3*
	*BB*	15.0 ± 3.5	14.7 ± 3.3	14.3 ± 3.4*	13.8 ± 3.5*	13.2 ± 3.4*	12.6 ± 3.5*	12.1 ± 3.4*	11.7 ± 3.3*
Ampli_SECOND_	*VL*	7.3 ± 2.3	7.5 ± 2.1	7.8 ± 2.4*	8.1 ± 2.6*	8.4 ± 2.5*	8.7 ± 2.5*	8.9 ± 2.6*	9.1 ± 2.8*
	*BB*	14.7 ± 3.8	15.1 ± 4.0	15.7 ± 4.1*	16.5 ± 4.3*	17.5 ± 4.2*	18.5 ± 4.5*	19.4 ± 4.2*	20.1 ± 4.0*
Dur_FIRST_	*VL*	11.4 ± 3.1	11.1 ± 2.9	10.8 ± 2.8*	10.4 ± 2.9*	9.9 ± 2.7*	9.6 ± 2.6*	9.3 ± 3.0*	9.1 ± 2.8*
	*BB*	10.2 ± 2.1	10.0 ± 2.1	9.8 ± 1.4*	9.5 ± 1.3*	9.1 ± 1.7*	8.8 ± 1.7*	8.5 ± 1.7*	8.3 ± 1.7*
Dur_SECOND_	*VL*	28.4 ± 9.8	27.8 ± 10.3	26.4 ± 9.3*	25.4 ± 9.2*	24.4 ± 9.2*	23.5 ± 8.9*	22.8 ± 8.8*	19.5 ± 8.2*
	*BB*	31.0 ± 10.3	30.2 ± 10.3	29.4 ± 9.6*	28.5 ± 9.3*	27.4 ± 9.4*	26.3 ± 9.2*	25.2 ± 9.4*	24.5 ± 9.5*

All values are expressed as mean ± SD

*Significantly different from control values (*P* < 0.05, *n* = 15)

## Discussion

The objective of the present study was to characterize the effects of brief muscle contractions (stimulated and voluntary) on the M-wave characteristics. The main findings were: (1) During the beginning of a tetanic stimulation we observed that Ampli_FIRST_ decreased, Ampli_SECOND_ increased, and M-wave duration decreased across the first five M waves of the train and then leveled off for the subsequent responses; (2) When a single electrical stimulus was superimposed on muscle contraction of increasing force level, we observed that Ampli_FIRST_ decreased, Ampli_SECOND_ increased, and M-wave duration decreased as force was raised from 0 to 70% MVC force.

### M waves at the beginning of a tetanic stimulation

It is well documented that, when continuous tetanic stimulation is applied, the muscle fibers shorten at the beginning of the stimulation due to the low stiffness of the tendon at low forces (Bennett et al. 1986; Shadwick 1990). As force develops further, the tendon’s stiffness increases rapidly, causing the muscle fibers to remain isometric (Ito et al. 1998). Therefore, the first few stimuli of a tetanic train would “test/catch” the muscle at progressively shorter lengths. McComas and Coworkers were the first to consider the possibility that the first M waves of a tetanic train could be “perturbed by a mechanical artifact” as the muscle shortens to its new length (McComas et al. 1994). Subsequently, Cupido et al. (1996) and Chang and Shields (2002) confirmed that the first M waves of a tetanic train underwent profound changes in amplitude and duration and attributed these changes to muscle shortening. Unfortunately, neither of these authors performed a detailed characterization of the changes in the M-wave profile at the onset of tetanic stimulation.

In agreement with the previous studies mentioned above, here we observed that the first ~ 5 M waves of the tetanic train exhibited notable differences in their amplitude and duration characteristics. Specifically, three distinct changes in the M-wave profile were observed from the first to the fifth response: a decrease in Ampli_FIRST_, an increase in Ampli_SECOND_, and a decrease in M-wave duration (see Fig. 2). The decrease in Ampli_FIRST_ and the increase in Ampli_SECOND_ were also observed in the *biceps brachii* M waves when muscle shortening was provoked by reducing the elbow joint angle (Fig. 1 of Brown et al. 1996). This coincidence further supports the idea that muscle shortening does occur during the first stimuli of a tetanic train.

The simultaneous adjustments in the M-wave profile observed at the beginning of a tetanic contraction (decreased Ampli_FIRST_, increased Ampli_SECOND_, and decreased duration) are in agreement with the predictions of EMG simulation models regarding the muscle shortening effects on M waves. (Mesin et al. 2006; Rodriguez-Falces et al. 2022). These models allow to rationalize the origin of each these adjustments. The primary cause for the decrease in Ampli_FIRST_ is muscle bulging (Mesin et al. 2006). Indeed, as the muscle fibers shorten in length and increase in diameter, the muscle bulges in thickness (Azizi et al. 2008; Dick and Wakeling 2018), which increases the distance from the fibers to the recording electrode, thus making Ampli_FIRST_ to decrease. The reason why muscle shortening provokes opposite effects on Ampli_SECOND_ and Ampli_FIRST_ is because, contrary to the first phase, the M-wave second phase is generated by the extinction of the action potentials at the tendon endings. Thus, as muscle fibers shorten, the tendon endings are moved closer to the recording electrode (located over the innervation zone), which allows a more synchronous arrival of the action potentials at the tendon, thus provoking an increase in Ampli_SECOND_. (Rodriguez-Falces et al. 2022). On the other hand, the decrease observed in the duration of the M wave is a natural consequence of the shorter length of the muscle fibers. Finally, an explanation must be offered for why the increase in Ampli_SECOND_ and the decrease in duration are so pronounced. The reason is that the shortened muscle fibers would show higher conduction velocities due to their increased fiber diameter (Hakansson 1956; Aidley 1998). Therefore, there are two factors (a decreased fiber length and an increased conduction velocity) acting in the same direction to cause an increase in Ampli_SECOND_ and a decrease in duration.

It could be argued that the M-wave changes observed at the beginning of the stimulation were caused mainly by an increase in the conduction velocity of muscle fibers, and that fiber shortening played a minor role. However, an increase in conduction velocity would cause an increase in both Ampli_FIRST_ and Ampli_SECOND_ (Rodriguez-Falces and Place 2017a), while a clear decline in Ampli_FIRST_ was observed in all participants. Thus, a shortening of the muscle, and the resulting bulging, is a necessary condition to produce the decrease in Ampli_FIRST_ (see above paragraph).

It is also important to rule out the possibility that the M-wave adjustments observed at the onset of the stimulation were due to fiber membrane depolarization and/or intracellular action potential fatigue. In this respect, we observed that the M-wave characteristics remained constant from the 6th stimulus to the 20th stimulus, thus indicating that fatigue was not an issue during the 1-s tetanic train. ####That M-wave profile did not vary during 1 s of tetanic stimulation is in agreement with the studies of Hanson (1974), who showed that at least 15–20 s of repetitive stimulation at 10 Hz was necessary to induce a detectable depression (and deformation) of the intracellular action potential.

### M waves superimposed on brief voluntary contractions of different intensities

It could be argued that, because tetanic stimulation is an “artificial” method of inducing muscle contractions (McComas et al. 1994), the muscle shortening process induced by a tetanic train is “artificial” and may lead to misleading changes in the M-wave profile. For this reason, we explored an alternative method of testing the muscle at different lengths by performing brief isometric voluntary contractions of varying force. That fascicle length is reduced as voluntary force increases from 0 to 60–90% MVC has been experimentally demonstrated in several muscles, such as the biceps brachii (Narici et al. 1996; Hodges et al. 2003).

As expected, we found that the M waves superimposed on voluntary contractions of different intensities showed marked differences in their amplitude and duration characteristics. Specifically, as voluntary force increased from 0 to 60–70% MVC force, we observed three main adjustments in the M-wave waveform: a decrease in Ampli_FIRST_, an increase in Ampli_SECOND_, and a decrease in M-wave duration (see Fig. 4). These adjustments in the M-wave profile were similar to those observed at the beginning of a tetanic train reported above, and to those found when the elbow joint angle was changed (Brown et al. 1996). It is important to note that the changes in Ampli_FIRST_, Ampli_SECOND_, and duration occurred rather linearly as contraction force was increased from 0 to 60% MVC force. This linear behavior fits reasonably well with the changes in fascicle length with muscle force in the gastrocnemius medialis (Narici et al. 1996) and *biceps brachii* (Hodges et al. 2003).

### The changes in the M-wave profile occurred up to a certain level of muscle force

The changes in the M-wave profile observed at the onset of a tetanic train occurred until the generated tetanic force reached ~ 70% of the maximal tetanic force. This indicates that muscle fibers’ length remains largely unchanged when tetanic force was increased above this force level. Similarly, we observed that the M waves superimposed on voluntary contractions undergo noticeable changes when force was increased up to ~ 70% MVC force. We believe that the primary cause for this upper force limit is that tendon’s stiffness increases with increasing force (Ito et al. 1998), and thus muscle fibers remain largely isometric when muscle force is increased beyond ~ 70% MVC force. It could be argued that, because in the tested muscles motor unit recruitment occurs up to ~ 60–70% MVC force (Gerdle et al. 1991; Bilodeau et al. 2003), the changes in the superimposed M wave with increasing force were partly due to the recruitment of additional (larger and faster) motor units. However, this factor (recruitment) does not play a role during tetanic stimulation. Besides, the M waves elicited during the voluntary contractions were supramaximal, and thus could only be marginally affected by motor unit recruitment. Moreover, the decrease in Ampli_FIRST_ cannot be explained by the recruitment of larger motor units.

### Adjustments in the M-wave profile to identify the occurrence of muscle shortening

The simultaneous combination of changes in the M-wave profile reported here (i.e., decreased Ampli_FIRST_, increased Ampli_SECOND_, and decreased duration) cannot be solely explained by alterations in fiber membrane properties or other neuromuscular propagation mechanisms. A summary of the evidence supporting that the occurrence of the three adjustments can only be due to muscle shortening are listed next: (1) They are grounded on biophysical principles of EMG signal generation (Rodriguez-Falces et al. 2022); (2) They are present in both simulated signals (Mesin et al. 2006) and in M waves recorded experimentally (present results); (3) They can be observed with two different methods of inducing muscle shortening, i.e. tetanic stimulation and voluntary contractions of different intensities (present results); (4) They are observed in fusiform muscles (*biceps brachii*) and also in muscles with pennate fibers (*vastus lateralis*). Therefore, when the three mentioned adjustments (decreased Ampli_FIRST_, increased Ampli_SECOND_, and decreased duration) occur simultaneously in the M wave, they can be taken as evidence that muscle shortening has occurred.

### Implications, limitations of the study and future research

The M wave is considered as a valuable tool for the assessment of the peripheral properties of the neuromuscular system. Indeed, researchers rely on the M-wave properties to investigate alterations in neuromuscular propagation that occur during/after muscle contractions as a result of muscle fatigue and/or changes in Na^+^–K^+^ pump activity (Hicks et al. 1989; Fuglevand et al. 1993; Fowles et al. 2002). However, here we have demonstrated that the M wave is greatly influenced by changes in the length of the muscle, an aspect that can vary during the course of an isometric contraction, even when such contraction is sustained at a constant force (Mademli and Arampatzis 2005). The dependence of the M-wave properties on muscle length represents an important limitation for the use of the M wave as an index of neuromuscular propagation: For example, how to differentiate whether a certain change in M wave amplitude is due to muscle shortening or to muscle fatigue? This problem can be partly mitigated: (1) if detailed knowledge of the muscle shortening effects on the M wave is available, and (2) by considering these shortening effects for the interpretation of the M-wave adjustments during/after a contraction.

We recently reported that during a sustained maximal voluntary contraction of 3 min of the quadriceps, the amplitude of the M-wave second phase increased initially for the first ~ 30 s and then decreased for the following ~ 60 s (Rodriguez-Falces and Place 2017b). We observed that the decline in Ampli_SECOND_ after 30 s of contraction occurred during the period when muscle force declines steeply, that is when the muscle is progressively increasing in length. Based on this, we propose that the decrease in Ampli_SECOND_ was caused by a lengthening of the muscle fascicles (and not by a reduction in membrane excitability due to fatigue), a possibility supported by the present results.

Another intriguing observation is the increase in M wave amplitude occurring immediately after a brief voluntary contraction, a phenomenon named as M-wave potentiation by McComas et al. (1994), which has been recently revisited by our group (Rodriguez-Falces et al. 2015; Rodriguez-Falces and Place 2017a). This augmentation of the M wave amplitude was interpreted by McComas et al (1994) as being due to an adaptative mechanism of the fiber membrane: the enhancement of the electrogenic Na^+^–K^+^ pump. However, we observed that the M wave enlargement only affected the second phase and, indeed, the first phase of the M wave slightly decreased after the contraction. Moreover, we observed that the enlargement of the second phase was systematically accompanied by a decrease in M-wave duration. These observations prompted us to propose that these M-wave adjustments were mainly due to the fact that muscle fibers remain shortened for a few seconds after a contraction (Rodriguez-Falces and Place 2017a).

The main limitation of the study was that changes in muscle length were not measured. Indeed, although we provided strong evidence indicating that our isometric protocols induced changes in muscle length, there was no direct proof that such length changes actually occurred, as we did not use ultrasound imaging. For future research it would be interesting to confirm the present findings using ultrasonography. Another practical aspect is the possibility that the skin slid over the muscle layer during the isometric contractions performed in the study cannot be excluded completely. However, the degree of skin sliding during isometric contractions is much lower than that during dynamic contractions, where the joint angle changes. Thus, we consider that the skin sliding effect could not have a big impact on the results. Another limitation is that only one repetition was performed for each test (tetanic stimulation or brief voluntary contraction). In this sense, repeating the tests would have increased the potential effect of fatigue.

## Conclusion

It has been shown that, during the beginning of a tetanic stimulation, Ampli_FIRST_ decreased, Ampli_SECOND_ increased, and M-wave duration decreased across the first five M waves of the train and then remained unchanged for the subsequent responses. These three adjustments in the M-wave profile were also observed when a single electrical stimulus was superimposed on muscle contraction of increasing force level.

The three adjustments in Ampli_FIRST_, Ampli_SECOND_, and duration reported here coincide with the simulation results obtained by previous EMG models, and these adjustments are well supported by biophysical principles of EMG signal generation. Two main implications emerged from this study: (1) The simultaneous occurrence of a decrease in Ampli_FIRST_, an increase in Ampli_SECOND_, and a decrease in M-wave duration can be taken as strong evidence that muscle shortening has taken place; (2) The present results are useful to differentiate the adjustments in the M wave caused by muscle shortening from those caused by muscle fatigue and/or changes in Na^+^–K^+^ pump activity.

## Data Availability

The datasets generated during and/or analysed during the current study are available from the corresponding author on reasonable request.
